# Association between the dynamics of the COVID-19 epidemic and ABO blood type distribution

**DOI:** 10.1017/S0950268821000030

**Published:** 2021-01-07

**Authors:** Yuefei Liu, Lisa Häussinger, Jürgen M. Steinacker, Alexander Dinse-Lambracht

**Affiliations:** 1 Division of Sports and Rehabilitation Medicine, Department of Internal Medicine II, University of Ulm, Ulm, Germany; 2 Center of Interdisciplinary Emergency Care, University of Ulm, Ulm, Germany

**Keywords:** ABO blood types, COVID-19, epidemic, infectious disease, pandemic, SARS-CoV-2

## Abstract

The coronavirus disease 2019 (COVID-19) pandemic is currently the most critical challenge in public health. An understanding of the factors that affect severe acute respiratory syndrome-coronavirus-2 (SARS-CoV-2) infection will help fight the COVID-19 pandemic. This study sought to investigate the association between SARS-CoV-2 infection and blood type distribution. The big data provided by the World Health Organization (WHO) and Johns Hopkins University were used to assess the dynamics of the COVID-19 epidemic. The infection data in the early phase of the pandemic from six countries in each of six geographic zones divided according to the WHO were used, representing approximately 5.4 billion people around the globe. We calculated the infection growth factor, doubling times of infection and death cases, reproductive number and infection and death cases in relation to the blood type distribution. The growth factor of infection and death cases significantly and positively correlated with the proportion of the population with blood type A and negatively correlated with the proportion of the population with blood type B. Compared with the lower blood type A population (<30%), the higher blood type A population (⩾30%) showed more infection and death cases, higher growth factors and shorter case doubling times for infections and deaths and thus higher epidemic dynamics. Thus, an association exists between SARS-CoV-2 and the ABO blood group distribution, which might be useful for fighting the COVID-19 pandemic.

## Introduction

The coronavirus disease 2019 (COVID-19) pandemic continues to rapidly increase worldwide, threatening public health and causing dramatic decreases in global economics and social life. According to the data derived from the World Health Organization (WHO) database, over 50 million confirmed COVID-19 cases and over 1.24 million deaths have occurred to date (reported on 08 November 2020), and new records for the daily numbers of new infection and death cases are steadily reported. This pandemic outbreak is difficult to control because factors affecting the COVID-19 epidemic are not thoroughly understood, and specific vaccines and treatments are still unavailable.

If one looks at the COVID-19 epidemic map provided by Johns Hopkins University (JHU) (e.g. the map picture on 15 May 2020), one would immediately recognise that quite a difference exists among the geographic districts with infections in the early phase of the pandemic. In fact, after the initial outbreak in China, the COVID-19 pandemic rapidly advanced to Europe and simultaneously to New York City, and afterwards, this pandemic spread to South America and East Mediterranean zones. An understanding of the factors with profound impacts on the pandemic is crucial for controlling the pandemic, and factors associated with the pandemic must be considered when making public policy and medical decisions. ABO blood types are associated with diverse infectious diseases, such as malaria, HIV and influenza [1–4]. Among the patients with confirmed COVID-19 cases who were treated in hospitals, the proportion of patients with blood type A was significantly higher than patients with blood type B [5], and furthermore, the severity and clinical outcomes of patients with COVID-19 patients were reported to be associated with blood types [6].

However, in these previous studies, a relatively small number of study subjects and/or subjects in a limited locality were investigated, and thus the data cover only a limited regional geographic zone and do not reveal the globally geographically uneven distribution of COVID-19 cases. Furthermore, the global dynamics of the COVID-19 epidemic were not investigated. We therefore conducted this big-data analysis of the association between the dynamics of the COVID-19 pandemic and ABO blood type distribution. The big data are derived from the official database provided by the WHO. The ABO blood type distribution serves as a typical genetic marker for the global geographic distribution of diverse diseases and public health issues. The determination of any factors that are associated with the COVID-19 epidemic might thus be important in fighting the COVID-19 pandemic.

For an epidemic of an infectious disease, the dynamic development of the infection is a determinant, which can be assessed by measuring several classic parameters, including the infection case growth factor (ICGF), infection case doubling time (IC-dt), reproductive number (RN), death case growth factor (DCGF) and death case doubling time (DC-dt). The difficulty in determining these parameters is the current high dynamics in infection development worldwide; thus, the endpoint of the total infection number remains unreached. Instead, we tried to assess these epidemic dynamics in the early exponential phase of the infection by assessing the infection cases and death cases according to the epidemical curves of the involved countries provided by JHU.

This study sought to investigate the relationship between the distribution of ABO blood types and the dynamics of the COVID-19 epidemic based on analyses of big data that cover the majority of the global population.

## Methods

### Data sources

For the analysis of the COVID-19 epidemic, the populations of six geographic regions divided according to the WHO were included: Africa, America, East Mediterranean, Europe, Southeast Asia and Western Pacific. Within each geographic zone, six countries were randomly selected for the analysis (Table 1 in the supplementary material). For randomisation, all countries in a geographic region were listed according to the first and second letters of each country name and then numbered in order. The randomisation was performed with SPSS software. However, at the time point of the analysis in the early epidemic phase, two countries, i.e. Botswana and Papua New Guinea, only reported a few COVID-19 cases that were not considered an epidemic, and therefore these countries were excluded from the analysis. Finally, the total population of the 34 countries was approximately 5 391 149 thousand (2016 WHO data).

Data on the blood type distribution in each country are derived from original research studies with respect to each corresponding country (Table 1 in the supplementary material). Since blood type A appeared more relevant in a previous correlation analysis and the mean proportion of individuals with blood type A in all 34 countries was 30%, the populations of all included countries was divided into higher (⩾30%) and lower (<30%) blood type A groups for the advanced analysis.

### Data analysis

**Fig. 1.**
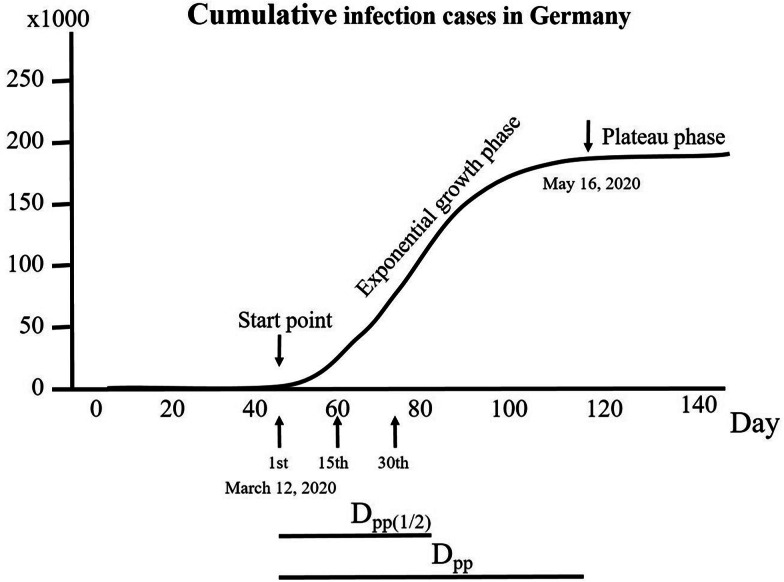
Illustration of mathematical analyses of the study parameters involving COVID-19 infection. As an example, the development of infection cases in Germany over 140 days is displayed to determine the points used for calculations. A curve with different phases can be identified from the course of cumulative infection cases. The 1st day was set as the day when the infection cases began to increase exponentially. In the early exponential phase, the infection growth factor (ICGF) was calculated. The death cases were obtained from the period between the 15th day after the start point and the 30th day. The plateau phase was reached when the course of cumulative infection cases no longer showed an exponential increase. The time interval (days) between the start point and the beginning of the plateau phase (Dpp) was calculated. Since a plateau phase was not yet reached in most countries, Dpp_(1/2)_ was set as half the mean Dpp and calculated for all countries.

The confirmed infection cases and death cases of each country were collected from the daily situation reports of the WHO. The start point (1st day) was set on the day when the infection cases began to increase exponentially to calculate the ICGF (1 + growth rate), and the corresponding infection cases were set as IC-begin. This point was identified on the curve provided by JHU by two independent coauthors. Taking Germany as an example, the day when the number of infection cases began to increase was 12 March 2020, with 1567 infection cases. The daily cases on the subsequent 14 consecutive days were input into SPSS^®^. The daily ICGF was calculated using the following formula [7] with SPSS software:
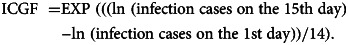
The RN was calculated using the methods described by the Robert-Koch-Institute (Cologne, Germany) [8, 9]. The calculation was based on the assumption of a viral generation time of 4 days and an incubation time of 5 days. The RN was calculated using the following formula:
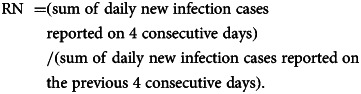
The death cases caused by or with COVID-19 were obtained from the daily situation reports of the WHO. Based on the clinical consideration that a death case occurred approximately 15 days after infection, the death cases on the 16th day and the following 14 consecutive days were input into SPSS^®^ to calculate the daily death case growth factor (DCGF, i.e. 1 + growth rate) that was calculated analogously to the ICGF as follows:
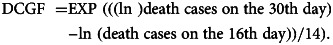
Furthermore, the doubling times for infection cases (IC-dt) and death cases (DC-dt) [10] were calculated using the following equations:
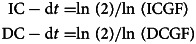
From the epidemic curve provided by JHU, a plateau phase for the infection cases was considered when the increase in new infection cases was no longer exponential, which was assessed for some countries. The time interval from the start point of the exponential phase to the beginning of the plateau phase was determined (days to the beginning of the plateau phase, Dpp). Furthermore, the numbers of cases on the day when the plateau phase was reached (nine countries up to 30 June 2020) were used to calculate the COVID-19 cases in the corresponding country. The mean Dpp was 51.2 days among these nine countries; thus, the Dpp_(1/2)_ was set at 26 days after the beginning of the exponential phase. Since most of the analysed countries did not reach their plateau phase in the early phase of the pandemic, the Dpp_(1/2)_ was determined for all analysed countries to count the infection and death cases (IC_Dpp(1/2)_ and DC_Dss(1/2)_, respectively). Figure 1 depicts all these methodological measures.

### Statistical analysis

In addition, a comparison of the epidemic dynamics was performed by dividing the population into groups with higher and lower proportions of individuals with blood type A either with a non-parametric (Mann−Whitney) test (Fig. 3a) or ANOVA (Fig. 3b), accordingly. Because the mean proportion of individuals with blood type A was 30%, the higher and lower blood type A groups were defined as ⩾30% or <30%, respectively.

The mathematical and statistical procedures were performed with SPSS^®^ (IBM, 25th edition, USA). A difference was assumed to be significant at *P* < 0.05.

## Results

For each parameter used to assess the epidemic dynamics, i.e. IC-begin, ICGF, DCGF, RN, IC-dt, DC-dt, IC_Dpp(1/2)_ and DC_Dss(1/2)_, an overall difference among the selected countries was observed using ANOVA (*P* < 0.01).

### Correlations between ABO blood groups and ICGF and DCGF

**Fig. 2.**
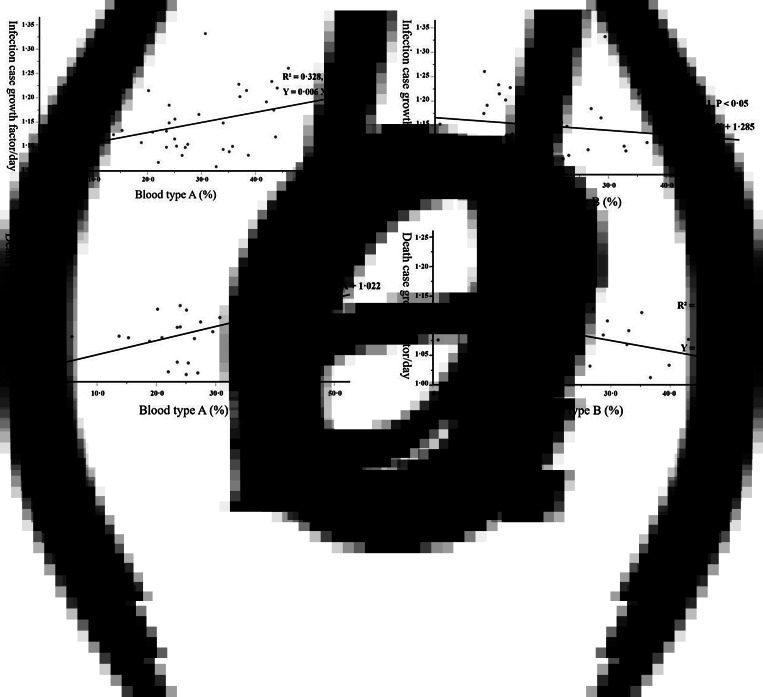
Correlations between the blood type distribution and COVID-19 infection case growth factor per day and death case growth factor per day.

ICGF correlated positively with the proportion of individuals with blood type A (*R*^2^ = 0.328, *P* < 0.05, Figure 2a) and negatively correlated with the proportion of individuals with blood type B (*R*^2^ = 0.210, *P* < 0.05. Figure 2b), but not with blood type O or AB.

Similarly, DCGF correlated positively with the proportion of individuals with blood type A (*P* < 0.01, Fig. 2c) and negatively with the proportion of individuals with blood type B (*P* < 0.05, Fig. 2d) but not with blood type O or AB.

### Infection case dynamics between the higher and lower blood type A groups

**Fig. 3.**
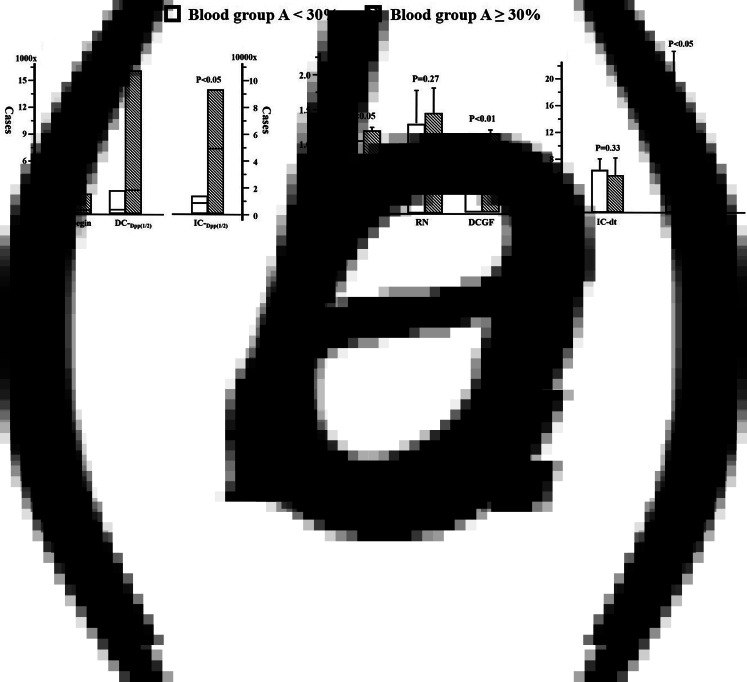
Comparison of the COVID-19 epidemic dynamics between the higher and lower blood type A populations. (a) IC-begin, infection cases on the first day when infection cases began to increase exponentially; DC_Dpp(1/2)_, death cases on 26th day after IC-begin; IC_Dpp(1/2)_, infection cases on 26th day after IC-begin. (b) ICGF, infection case growth factor per day; RN, reproductive number; DCGF, death case growth factor per day. (c) IC-dt, infection case doubling time per day; DC-dt, death case doubling time per day.

At the beginning of the exponential phase of the COVID-19 epidemic, a significant difference in infection cases was not observed between the higher and lower blood type A groups (*P* > 0.05, Fig. 3a). At Dpp_(1/2),_ the number of infection cases was distinctly higher in the group with a higher blood type A proportion than in the group with a lower blood type A proportion (*P* < 0.05).

During the first 15 days of the exponential growth phase, ICGF was significantly higher in the group with a higher blood type A proportion than in the group with a lower blood type A proportion (*P* < 0.05, Fig. 3b). IC-dt was reciprocally shorter in the higher blood group A group than in the lower blood type A group (*P* < 0.05, Fig. 3c). Although the reproductive number was slightly higher in the higher blood type A group than in the lower blood group A group, this difference was not statistically significant (*P* > 0.05, Fig. 3b).

### DCGF dynamics between higher and lower blood type A groups

The dynamic changes in death cases indicated by DCGF showed a significant difference between both groups, i.e. higher in the group with a higher blood type A proportion (*P* < 0.01, Fig. 3b). Similarly, the DC_Dpp(1/2)_ results showed more deaths in the higher blood type A group than in the lower blood type A group (*P* < 0.05). Accordingly, the death case doubling time was significantly shorter in this group (*P* < 0.05, Fig. 3c).

## Discussion

The COVID-19 pandemic is challenging public health care. Classically, four major measures are essential to control an epidemic disease: controlling the disease origin, cutting the infectious transmission pathway, effectively treating patients and improving herd immunity. However, to date, the origin of the COVID-19 epidemic remains uncertain, a clinically proven vaccine to improve immunity against the COVID-19 epidemic is still unavailable, and an effective therapy for SARS-CoV-2-infected patients has not yet been established. Therefore, the unique certainly effective measure available at present is to disrupt the widespread transmission, i.e. personal protection measures, activity restrictions and community lockdowns. Unfortunately, lockdown causes a series of adverse effects, e.g. a decrease in the community economy, reduction in productivity, restriction of people's freedom and hampering public health care. Therefore, the ability to rationally balance the epidemic dynamics and lockdown is of critical social and health interest, based on the observation and analysis of the dynamics of the COVID-19 epidemic.

Indeed, the ability to determine the dynamics of the COVID-19 epidemic is by no means an easy task because a number of factors exert profound effects on these dynamics. Exact data on infection and death cases are difficult to obtain due to limited manpower and health care resources in testing the population, not to mention limited test certainty in terms of sensitivity and specificity, various incubation times, different social environments and activities etc. Therefore, an analysis based on big data appears to be a reasonable method to assess the dynamics of the COVID-19 epidemic globally. Currently, the WHO and JHU are monitoring these dynamics and providing useful information; therefore, we used these useful tools and performed further analyses with a specific focus on genetic factors. Furthermore, with the advanced and wider spread of the COVID-19 epidemic, factors including changes in the social behaviour of the people, restrictions on social activity and increasing herd immunity will significantly modify the dynamics of the epidemic. Therefore, we focused the analysis on the early phase of the pandemic when the pandemic dynamics were more ‘natural’. Indeed, the IC-begin data did not show a statistically significant difference between the higher and lower blood type A groups.

In fact, genetic factors play an important role in many diseases, such as cardiovascular diseases [11], diabetes mellitus [12], cancers [13] and infectious diseases, including malaria [14], HIV and influenza [15, 16]. As a genetic factor, the ABO blood type distribution has been extensively investigated in the field of infectious diseases [15–17]. Meanwhile, interest in investigating the relationship between the ABO blood system and the COVID-19 epidemic is increasing [6, 18, 19]. The first study suggested that among infected individuals, the proportion of patients with blood type A was higher [19]. In this study, the researchers analysed 2173 patients with confirmed COVID-19 cases who were treated in hospitals and found a higher proportion of blood group A in these patients than in a comparable population without COVID-19. Since a small number of study subjects from the same locality were analysed, the data were quite limited. Recently, Ellinghaus *et al*. reported a larger multicentre study and documented a distinct association between ABO blood types and the severity of COVID-19 cases [6]. Unfortunately, in their study, the dynamics of the COVID-19 epidemic were not investigated. We thus conducted this study based on the globally available big data to ascertain an association between the distribution of ABO blood types and the dynamics of the COVID-19 epidemic.

The results of our analyses showed a positive correlation between the proportion of blood group A in the global population and the dynamics of the COVID-19 epidemic, as reflected by the infection case growth factor, and a negative correlation between the blood group B proportion and the infection dynamics (Fig. 2). Furthermore, we obtained similar results for the relationship between blood group types and the death case growth factor (Fig. 2). In this context, our results are consistent with these aforementioned studies and strongly confirm the association between the distribution of blood group types and the COVID-19 epidemic. The lack of significant differences in RN and IC-dt between both groups might be attributed to the method. RN represents the change in new infection cases in the previous 8 days and might be less dynamic as the infection cases change. IC-dt is only a parameter used by our group, and further studies are needed to clarify whether this parameter is suitable for assessing the COVID-19 epidemic; thus, the results should be interpreted with caution.

Based on the results described above, we divided the global population into two groups according to the proportion of individuals with blood type A, i.e. the higher blood type A group (⩾30%) and the lower group (<30%), and compared the differences in the epidemic dynamic parameters between both groups. As shown in Figure 3, with the exception of the infection cases at the beginning of the exponential phase and the reproductive number, all other parameters were significantly higher in the higher blood type A group than in the lower blood type A group. These results further reveal an association between the blood type distribution and the epidemic dynamics of SARS-CoV-2 infection. We also analysed the relationships between the population life expectancy as well as health care expenses and COVID-19 cases (data not shown) based on data provided by the WHO. With an increase in life expectancy, the number of COVID-19 cases per 100 000 people increased, suggesting a higher SARS-CoV-2 infection susceptibility in older people, which has already been confirmed. A significant correlation was not observed between health care expenses and COVID-19 epidemic dynamics in the analysed countries.

The mechanisms responsible for this association have yet to be explored. Currently, SARS-CoV-2 infection is presumed to begin with the docking of its spiking protein to ACER2 [20–22]. Researchers have not clearly determined whether the ACER2 expression level differs among individuals with different blood types and whether people with blood type A have higher ACER2 expression levels. Certainly, further studies are necessary to explore the mechanisms.

The consideration of the association between the COVID-19 epidemic and blood type distribution may be important and meaningful to make reasonable decisions in combating the COVID-19 pandemic among different people with respect to their blood type distribution. In general, countries with a lower proportion of individuals with blood type A have a lower economic status and poorer health care resources, which might be exaggerated by community lockdown. A reasonable approach for these countries might be to implement slower, later or milder restriction measures due to the lower dynamics of the COVID-19 epidemic, whereas the restriction measure to combat the COVID-19 epidemic in populations with a higher proportion of people with blood type A ought to be implemented more actively and earlier.

Certainly, the association between ABO blood groups and the dynamics of the COVID-19 epidemic does not mean that ABO blood groups are unique and determining factors affecting the COVID-19 epidemic. Other factors, including population age, efficacy of the health care system and socioeconomic status, also have profound impacts on this epidemic. Furthermore, the results of this study should be interpreted with great caution since the available big data might be influenced by the capacity to diagnose, trace, treat and report cases.

## Supplementary material

For supplementary material accompanying this paper visit https://doi.org/10.1017/S0950268821000030. click here to view supplementary material

## Data

The data that support the findings of this study are openly available at https://www.who.int/emergencies/diseases/novel-coronavirus-2019/situation-reports and https://coronavirus.jhu.edu/map.html.

## References

[1] Damena D (2019) Genome-wide association studies of severe *P. falciparum* malaria susceptibility: progress, pitfalls and prospects. BMC Medical Genomics 12, 120–134. doi:10.1186/s12920-019-0564-x. 31409341 PMC6693204

[2] Degarege A (2019) Effect of the ABO blood group on susceptibility to severe malaria: a systematic review and meta-analysis. Blood Reviews 33, 53–62. 30029997 10.1016/j.blre.2018.07.002

[3] Siransy LK (2015) ABO/Rh blood groups and risk of HIV infection and hepatitis B among blood donors of Abidjan, Cote D'ivoire. European Journal of Microbiology and Immunology 5, 205–209. 26495131 10.1556/1886.2015.00029 PMC4598888

[4] Horby P (2012) The role of host genetics in susceptibility to influenza: a systematic review. PLoS One 7, e33180. 22438897 10.1371/journal.pone.0033180 PMC3305291

[5] Wu Y (2020) Relationship between ABO blood group distribution and clinical characteristics in patients with COVID-19. Clinica Chimica Acta 509, 220–223. 10.1016/j.cca.2020.06.026 PMC7832938 32562665

[6] Ellinghaus D (2020) Genomewide association study of severe covid-19 with respiratory failure. New England Journal of Medicine **383**:1522–15341. 10.1056/NEJMoa2020283 PMC7315890 32558485

[7] e Fernandes T (2020) Chaotic model for COVID-19 growth factor. Research on Biomedical Engineering. doi:10.1007/s42600-020-00077-5.

[8] an der Heiden M and Hamouda O (2020) Schätzung der aktuellen entwicklung der SARS-Cor-2-epidemie in deutschland - nowcasting. Epidemiologisches Bulletin 17, 10–6.

[9] Zietz M, Zucker J and Tatonetti NP (2020) Association between blood type and COVID-19 infection, intubation, and death. Nature Communications **11:** 5761–5766. 10.1038/s41467-020-19623-x PMC7666188 33188185

[10] Patel SB and Patel P (2020) Doubling time and its interpretation for COVID 19 cases. Statistical Update pISSN 0976 3325 /eISSN 2229 2816.

[11] Capuzzo E (2016) The relationship between ABO blood group and cardiovascular disease: results from the cardiorisk program. Annals of Translational Medicine 4, 189–194. 27294085 10.21037/atm.2016.03.58 PMC4885887

[12] Williams DR and Cartwrigth RA (1979) Genetic polymorphisms in diabetics and non-diabetics. Journal of Medical Genetics 16, 351–357. 117108 10.1136/jmg.16.5.351 PMC1012608

[13] Antwi SO (2018) Pancreatic cancer risk is modulated by inflammatory potential of diet and ABO genotype: a consortia-based evaluation and replication study. Carcinogenesis 39, 1056–1067. 29800239 10.1093/carcin/bgy072 PMC6067129

[14] Alemu G and Mama M (2016) Assessing ABO/Rh blood group frequency and association with asymptomatic malaria among blood donors attending Arba Minch blood bank, south Ethiopia. Malaria Research and Treatment. doi:10.1155/2016/804376. PMC4748098 26925291

[15] Tyrrell DA, Sparrow P and Beare AS (1968) Relation between blood groups and resistance to infection with influenza and some picornaviruses. Nature 220, 819–820. 4301643 10.1038/220819a0

[16] Davison GM, Hendrickse HL and Matsha TE (2020) Do blood group antigens and the red cell membrane influence human immunodeficiency virus infection? Cells 9, 845–855. 10.3390/cells9040845 PMC7226767 32244465

[17] Evans AS, Shepard DA and Richards VA (1972) ABO blood groups and viral diseases. Yale Journal of Biology and Medicine 45, 81–92. PMC2591859 4336480

[18] Zaidi FZ (2020) COVID-19 and the ABO blood group connection. Transfusion *and* Apheresis Science **59:** D: DOI: 10.1016/j.transci.2020.102838 PMC7834841 32513613

[19] Zhao J (2020) Relationship between the ABO blood groups and the covid-19 susceptibility. Clinical Infectious Diseases. doi: ciaa1150, 10.1093/cid/ciaa1150. PMC7454371 32750119

[20] Guillon P (2003) Inhibition of the interaction between ths SARS-CoV spike protein and its cellular receptor by anti-histo-blood group antibodies. Glyconbiology 18, 1085–1093. 10.1093/glycob/cwn093 PMC7108609 18818423

[21] Wan Y (2020) Receptor recognition by novel coronavirus from Wuhan: an analysis based on decade-long structural studies of SARS. Journal of Virology 94:doi:10.1128/JVI.00127-20. PMC7081895 31996437

[22] Hoffmann M, (2020) SARS-CoV-2 cell entry depends on ACE2 and TMPRSS2 and is blocked by a clinically proven protease inhibitor. Cell 181, 271–280. 32142651 10.1016/j.cell.2020.02.052 PMC7102627

