# Supplementary material for: Association between the dynamics of the COVID-19 epidemic and ABO blood type distribution
Source: Epidemiol Infect. 2021 Jan 7;149:e19. doi: 10.1017/S0950268821000030 (PMC7844181; doi:10.1017/S0950268821000030)
Supplement: Supplementary file 1 [file S0950268821000030sup001.docx]

Table 1. The population and blood type distribution of the selected countries for the study analyses* [^1-27]^.

|  |  |  |  | Blood | type |  |
| --- | --- | --- | --- | --- | --- | --- |
| Region | Country | Population in thousands | A (%) | B (%) | O (%) | AB (%) |
| Africa | Algeria | 40606 | 20,93 | 12,30 | 66,77 | ,00 |
|  | Cameroon | 23439 | 25,07 | 21,86 | 48,62 | 4,45 |
|  | Dem Rep Congo | 78736 | 26,97 | 20,23 | 49,11 | 3,69 |
|  | Ethiopia | 102403 | 32,70 | 20,90 | 42,10 | 4,30 |
|  | South Africa | 56015 | 35,73 | 19,02 | 39,35 | 5,85 |
| America | Brazil | 207653 | 34,00 | 8,00 | 36,00 | 2,50 |
|  | Canada | 36290 | 42,00 | 9,00 | 46,00 | 3,00 |
|  | Ecuador | 16385 | 15,29 | 13,36 | 69,46 | 1,97 |
|  | Mexico | 127540 | 27,44 | 8,93 | 61,82 | 1,81 |
|  | Peru | 31774 | 5,75 | ,86 | 93,10 | ,30 |
|  | United States | 322180 | 37,10 | 12,20 | 46,60 | 4,10 |
| Eastern Mediterranean | Egypt | 95689 | 35,12 | 23,12 | 31,94 | 9,74 |
|  | Iran | 80277 | 29,50 | 28,70 | 33,90 | 7,90 |
|  | Israel | 8192 | 36,90 | 13,00 | 46,30 | 3,70 |
|  | Pakistan | 193203 | 23,50 | 33,00 | 35,40 | 8,00 |
|  | Saudi Arabia | 32276 | 26,40 | 17,70 | 51,30 | 4,30 |
|  | United Arab Emirates | 9270 | 24,00 | 22,80 | 48,40 | 4,70 |
| Europe | Germany | 81915 | 43,00 | 11,00 | 41,00 | 5,00 |
|  | Russia | 143965 | 34,00 | 20,00 | 40,00 | 6,00 |
|  | Spain | 46348 | 46,07 | 8,54 | 41,61 | 3,78 |
|  | Sweden | 9838 | 43,70 | 11,10 | 40,10 | 5,00 |
|  | Turkey | 79512 | 44,00 | 16,20 | 33,30 | 6,50 |
|  | United Kingdom | 66789 | 43,36 | 8,44 | 45,40 | 3,03 |
| South east Asia | Bandgladesh | 162952 | 23,50 | 39,80 | 27,60 | 9,20 |
|  | India | 1324171 | 18,85 | 32,69 | 38,75 | 5,27 |
|  | Indonesia | 261115 | 13,70 | 43,06 | 39,27 | 3,97 |
|  | Myanmar | 52855 | 25,02 | 36,65 | 31,67 | 6,67 |
|  | Sri Lanka | 20798 | 22,00 | 19,50 | 53,70 | 4,90 |
|  | Thailand | 68864 | 20,19 | 35,19 | 37,65 | 6,97 |
| Western Pacific | Australia | 24126 | 38,32 | 11,12 | 47,79 | 2,78 |
|  | China | 1411415 | 30,70 | 29,40 | 30,20 | 9,70 |
|  | Japan | 127749 | 38,65 | 22,15 | 29,25 | 9,95 |
|  | Malaysia | 31187 | 24,00 | 27,00 | 40,00 | 9,00 |
|  | Singapore | 5622 | 25,37 | 26,46 | 41,92 | 6,25 |

* Blood type distribution was statistically different among the countries by ANOVA

References

1. Adiputra IN. Distribution of ABO blood groups in Bali. Journal of Human (Tokyo) 1977;**6**: 206-208.

2. Ben Q, Liu J, Wang W, Guo F, Yao W, Zhong J and Yuan Y. Association between ABO blood types and sporadic pancreatic neuroendocrine tumors in the Chinese Han population. *Oncotarget* 2017;**8**: 54799-54808.

3. Canizalez-Roman A, Campos-Romero A, Castro-Sanchez JA, Lopez-Martinez MA, Andrade-Munoz FJ, Cruz-Zamudio CK, et al. Blood Groups Distribution and Gene Diversity of the ABO and Rh (D) Loci in the Mexican Population. *Biomed Research International* 2018: <https://doi.org/10.1155/2018/1925619>.

4. Chen XH, Deng WM, Zou L, Huang XR, Lu BX and Zhang XL. [Correlation of ABO groups to hypertensive intracerebral hemorrhage]. *Nan Fang Yi Ke Da Xue Xue Bao* 2010;**30**: 2521-2522.

5. DOBSON AM and Ikin EW. The ABO blood groups in the United Kingdom; frequencies based on a very large sample. *Journal of Pathology and Bacteriology* 1946;**58**: 221-227.

6. Dubinski D, Won SY, Behmanesh B, Brawanski N, Geisen C, Seifert V, Senft C and Konczalla J. The clinical relevance of ABO blood type in 100 patients with acute subdural hematoma. *PLoS One* 2018;**4**: e0204331.

7. Egawa N, Lin Y, Tabata T, Kuruma S, Hara S, Kubota K and Kamisawa T. ABO blood type, long-standing diabetes, and the risk of pancreatic cancer. *World Journal of Gastroenterology* 2013;**19**: 2537-2542.

8. Fowkes FJ, Michon P, Pilling L, Ripley RM, Tavul L, Imrie HJ, et al. Host erythrocyte polymorphisms and exposure to Plasmodium falciparum in Papua New Guinea. *Malaria Journal* 2008;7: doi: 10.1186/1475-2875-7-1.

9. Fujita Y, Tanimura M and Tanaka K. The distribution of the ABO blood groups in Japan. *Jinrui Idengaku Zasshi* 1978;**23**: 63-109.

10. Gasso P, Ritter MA, Mas S and Lafuente A. Influence of ABO genotype and phenotype on angiotensin-converting enzyme plasma activity. *Journal of the*Renin*-*Angiotensin*-*Aldosterone System 2014;**15**: 580-584.

11. Goedde HW, Hirth L, Benkmann HG, Pellicer A, Pellicer T, Stahn M and Singh S. Population genetic studies of serum protein polymorphisms in four Spanish populations. II. *Human Heredity* 1973;**23**: 135-146.

12. Gotsman I, Keren A, Zwas DR, Lotan C and Admon D. Clinical Impact of ABO and Rhesus D Blood Type Groups in Patients With Chronic Heart Failure. *American Journal of Cardiology* 2018;**122**: 413-419.

13. Hall RL. Authropometric and genetic studies in a Russian Old Believer population. *Human Heredity* 1973;**23**: 113-122.

14. Hatt D and Parsons PA. Association between surnames and blood groups in the Australian population. *Acta Genetica et Statistica medica* 1965;**15**: 309-318.

15. Kang JF and Zhang LD. [Investigation relationship between chronic benzene poisoning and ABO blood type]. *Zhonghua Lao Dong Wei Sheng Zhi Ye Bing Za Zhi* 2011;**29**: 781-782.

16. Kurexijiang T, Hamulati W, Nuermaimaiti Y, Muyasaier K, Palida HR, Halike Y, et al. [Comparative investigation of the Rh blood type distribution between the Uygur and Han nationalities in the Khotan area of Xinjiang Autonomous Region]. *Di Yi Jun Yi Da Xue Xue Bao* 2004;**24**: 447-449.

17. Linn S, Schoenbaum SC and Lieberman E. ABO and rhesus blood groups and adverse outcomes of pregnancy. *Human Heredity* 1993;**43**: 366-370.

18. Matson GA, Sutton HE, Swanson J and Robinson A. Distribution of hereditary blood groups among Indians in South America. II. In Peru. *American Journal of Physical Anthropology* 1966;**24**: 325-349.

19. Mya-Tu M, May MY and Thin TH. Blood groups of the Burmese population. *Human Heredity* 1971;**21**: 420-430.

20. Nabil B, Bounab S, Benazzi L and Yahiaoui M. Genetic polymorphisms of blood donors in Algeria through blood groups ABO, RH, and Kell. *Transfusion Clinique et Biologigue* 2020;**27**: 43-51.

21. Ndoula ST, Noubiap JJ, Nansseu JR and Wonkam A. Phenotypic and allelic distribution of the ABO and Rhesus (D) blood groups in the Cameroonian population. *Internation Journal Immunogenetics* 2014;**41**: 206-210.

22. Pelzer U, Klein F, Bahra M, Sinn M, Dorken B, Neuhaus P, et al. Blood group determinates incidence for pancreatic cancer in Germany. *Frontiers in Physiology* 2013;**4**: doi: 10.3389/fphys.2013.00118. eCollection 2013.

23. Peng YF, Goyal H, Lin H, Liu DC and Li L. Serum amylase activity altered by the ABO blood group system in Chinese subjects. *Journal of Clinical Laboratory Analysis* 2019;**33**: doi: [10.1002/jcla.22883](https://dx.doi.org/10.1002%2Fjcla.22883)

24. Rihmer Z and Arato M. ABO blood groups in manic-depressive patients. *Journal of Affective Disorders* 1981;**3**: 1-7.

25. Rinieris P, Rabavilas A, Lykouras E and Stefanis C. Neuroses and ABO blood types. *Neuropsychobiology* 1983;**9**: 16-18.

26. WEINER JS and ZOUTENDYK A. Blood-group investigation on Central Kalahari Bushmen. *Nature* 1959;**183**: 843-844.

27. Zakai NA, Judd SE, Alexander K, McClure LA, Kissela BM, Howard G, et al. ABO blood type and stroke risk: the REasons for Geographic And Racial Differences in Stroke Study. *Journal of Thrombosis and Haemostasis* 2014;**12**: 564-570.
